# CD93 is Associated with Glioma-related Malignant Processes and Immunosuppressive Cell Infiltration as an Inspiring Biomarker of Survivance

**DOI:** 10.1007/s12031-022-02060-4

**Published:** 2022-08-25

**Authors:** Kaiming Ma, Suhua Chen, Xin Chen, Xiaofang Zhao, Jun Yang

**Affiliations:** 1 Department of Neurosurgery, Peking University Third Hospital, Haidian District, 49 North Garden Rd, Beijing, 100191 China; 2 Center for Precision Neurosurgery and Oncology of Peking University Health Science Center, Beijing, China

**Keywords:** CD93, Biomarker, Glioma, Prognosis, Immunosuppression, Target

## Abstract

Previous reports have confirmed the significance of CD93 in the progression of multiple tumors; however, there are few studies examining its immune properties for gliomas. Here, we methodically investigated the pathophysiological characteristics and clinical manifestations of gliomas. Six hundred ninety-nine glioma patients in TCGA along with 325 glioma patients in CGGA were correspondingly collected for training and validating. We analyzed and visualized total statistics using RStudio. One-way ANOVA and Student’s *t*-test were used to assess groups’ differences. All differences were considered statistically significant at the level of *P* < 0.05. CD93 markedly upregulated among HGG, MGMT promoter unmethylated subforms, IDH wild forms, 1p19q non-codeletion subforms, and mesenchyme type gliomas. ROC analysis illustrated the favorable applicability of CD93 in estimating mesenchyme subform. Kaplan–Meier curves together with multivariable Cox analyses upon survivance identified high-expression CD93 as a distinct prognostic variable for glioma patients. GO analysis of CD93 documented its predominant part in glioma-related immunobiological processes and inflammation responses. We examined the associations of CD93 with immune-related meta-genes, and CD93 positively correlated with HCK, LCK, MHC I, MHC II, STAT1 and IFN, while adverse with IgG. Association analyses between CD93 and gliomas-infiltrating immunocytes indicated that the infiltrating degrees of most immunocytes exhibited positive correlations with CD93, particularly these immunosuppressive subsets such as TAM, Treg, and MDSCs. CD93 is markedly associated with adverse pathology types, unfavorable survival, and immunosuppressive immunocytes infiltration among gliomas, thus identifying CD93 as a practicable marker and a promising target for glioma-based precise diagnosis and therapeutic strategies.

**Supplementary Information:**

The online version contains supplementary material available at 10.1007/s12031-022-02060-4.

## Introduction

Gliomas are intractable idiopathic neoplasms that comprise the largest proportion of the central nervous system (CNS) (Louis et al. 2021; Ostrom et al. 2020). The latest research reported that the incidence of gliomas reached 80.8% of the overall incidence of CNS essential malignancy, while the death toll from gliomas was 88.1% of the total fatalities due to CNS neoplasms (Ostrom et al. 2020). For such complex neoplasms, the currently approved clinical therapeutic protocols include surgery remedies, chemotherapeutic drugs, radiotherapeutics, and also several burgeoning adjunctive therapeutics such as molecule-targeted therapeutics, immunological approaches, and tumor treating fields (TTFs) (Nabors et al. 2020; Xu et al. 2020). Nonetheless, these therapeutics alone or in combination are still unsatisfactory after verification with clinical practical application, notably ameliorating the overall prognosis for patients diagnosed with gliomas, particularly for high-grade gliomas (HGG) (Ma et al. 2021; Tan et al. 2020; Xu et al. 2020). The most recent version of the World Health Organization classification of CNS tumors reaffirmed that molecular biomarkers are essential for providing practical evidence for glioma-based diagnosis and healing (Louis et al. 2021). Therapeutic strategies that integrate more precise glioma-related biomarkers possess the potential to surmount existing therapeutic woes (Tan et al. 2020). Hence, identifying more precise biomarkers that are inextricably linked to glioma clinicopathology and progression is imperative (Ma et al. 2022).

CD93, corresponding to complement component C1q receptor(C1qRp), acts as a type I transmembrane glycoprotein with a calcium-dependent carbohydrate-binding domain from the C-type superfamily of lectins (Borah et al. 2019; Steinberger et al. 2002). It is formed by one single spaning area, one endocellular domain, one C‐type lectin‐like domain, one mucin domain together with five epidermal growth factor (EGF)‐like domains (Greenlee et al. 2008; McGreal and Gasque 2002; Petrenko et al. 1999). CD93 has two types: cell-associated full-length CD93 and abridged soluble CD93 (sCD93) (Greenlee-Wacker et al. 2011). sCD93 is the enzymolysis cleaved extracellular domain of transmembrane CD93 (Bohlson et al. 2005; Greenlee et al. 2009), which predominantly riched in the extracellular space and circulation (Strawbridge et al. 2016; Turk et al. 2001). Under physiological conditions, CD93 is mainly riched in endothelial cells (ECs), maturing B cells, circulating myeloid cells, platelets and some hematopoietic subsets such as hematopoietic stem cells (Blackburn et al. 2019; Fonseca et al. 2001; Greenlee et al. 2009; Nepomuceno and Tenner 1998). The major physiological function of CD93 is regulating angiogenesis (Galvagni et al. 2016; Langenkamp et al. 2015; Lugano et al. 2018). It involves in several physiological processes such as the migration and adhesion of ECs, the extravasation of leukocytes, cell apoptosis, innate immunity, inflammation and the remodeling of extracellar matrix (ECM) (Greenlee et al. 2009; Harhausen et al. 2010; Lugano et al. 2020, 2018; Sigari et al. 2016).

Regarding pathological processes, CD93 is primarily located in ECs of new blood vessels in various neovascularization pathologies (Galvagni et al. 2017; Langenkamp et al. 2015; Tosi et al. 2017). Previous explorations have shown that CD93 is significantly upregulated and plays an important role in tumor vasculatures of renal cell carcinomas (Masiero et al. 2013), pancreatic adenocarcinoma (Sun et al. 2021b), nasopharyngeal carcinoma (Bao et al. 2016) and colon cancer (Olsen et al. 2015), which made it a probable antiangiogenic target for these cancers (Barbera et al. 2021; Orlandini et al. 2014; Sun et al. 2021b). Sustained vascularization and immunomodulation are two interconnected hallmarks of cancer ascribed to glioma (Mosteiro et al. 2022; Torrisi et al. 2022). The abnormal structure of the tumor vasculature and restricted blood perfusion prevent immune cells from infiltrating tumors efficiently, which results in an unbalanced and immunosuppressive tumor microenvironment (TME) (Jain 2014). Recent reports have confirmed that CD93 is overexpressed in glioblastoma (GBM) vasculature both in mRNA (Xie et al. 2021) and protein level compared with low-grade gliomas (LGG) and normal brain tissue (Lugano et al. 2018). CD93 has been verified as a crucial regulator in modulating abnormal angiogenesis, vascular function, cytoskeleton orchestrating, ECM organization and glucometabolic regulation in GBM (Langenkamp et al. 2015; Lugano et al. 2018; Strawbridge et al. 2016). The expression level of CD93 was proved to be associated with the prognosis of patients with HGG (Langenkamp et al. 2015). These findings identify CD93 as a possible therapy target for gliomas (Langenkamp et al. 2015; Lugano et al. 2018). However, previous studies have primarily focused on GBM, and no large-scale clinical analysis of cases has revealed the comprehensive characteristics of CD93 in the context of gliomas. Moreover, there are few studies examining the immune properties of gliomas, particularly in the context of immunosuppressive processes, thus making it difficult for us to objectively recognize its role and deeply restricting the clinical translation of CD93-targeted therapies for glioma.

Here, we methodically investigated CD93 in the context of glioma from its expression patterns, pathobiological roles, and prognosis, particularly the characteristics of glioma-relevant immunosuppressive responses together with immunocyte infiltration degrees. A total of 699 patients diagnosed with gliomas from the Cancer Genome Atlas (TCGA) along with 325 glioma patients from the Chinese glioma genome atlas (CGGA) were correspondingly collected for the training set and validation set based on ribonucleic acid sequencing (RNA-seq) data. Our exploration provides strong practical evidence for the CD93-targeted tactics of glioma-based precise diagnosis and therapies.

## Materials and Methods

### Patients and Samples

A total of 1024 patients diagnosed with WHO grade II-IV gliomas were included in this study. Among them, 699 glioma patients in the TCGA set were classified as the training set, while the other 325 glioma patients in the CGGA set were classified as the validation set. We downloaded the total RNA-seq data, molecular pathology information, and survival time of former training sets from the website http://cancergenome.nih.gov/, while the corresponding data for the latter validation set were obtained from http://www.cgga.org.cn. Seventy-eight glioma patients who lacked intact data were removed after primary assessment, and these included 63 patients in the training set and 15 patients in the validation set. We obtained approval (counterpart number: S2020018) from the Ethics Committees of Peking University Third Hospital.

### Statistical Analysis

Statistical analyses together with figure visualization were performed using RStudio with corresponding application packages such as “survival,” “survminer,” “ggplot2,” “pROC,” “pheatmap,” “devtools,” “corrplot,” “ggpubr”, and “corrgram” that were obtained from the website http://www.r-project.org. Logarithmic transformations were applied to the transcriptome sequencing data that were analyzed in this study prior to further analysis. Kaplan–Meier survivorship curvilinear analyses together with multivariable Cox analyses were performed to compare survival differences among the included patients. Spearman correlation analyses were used for sequencing and for sifting genes that were markedly related to CD93. Pearson’s association analyses were similarly applied for correlational degree assessments. Gene ontology function analysis of gene biological processes and molecular functions together with cellular components was conducted via the website of DAVID Bioinformatical Resource (https://david.ncifcrf.gov/). AmiGO2 version 2.5.17 was utilized for downloading analyzing immunogene subsets to investigate the functions of CD93 among glioma-associated immunity responses (http://amigo.geneontology.org/amigo). Single factor variance analyses were applied for difference testing using no fewer than three statistical clusters, while the difference testing for each of the two statistical clusters was completed using Student’s *t*-test. A *P* value of less than 0.05 was considered to be statistically significant.

## Results

### CD93 is Indicated to be Markedly Upregulated Among High Grade, Isocitrate Dehydrogenases (IDH) Wild Form, Non-codeleted 1p/19q Subform in Combination with Unmethylated O^6^-Methylguanine-Deoxyribonucleic Acid Methyltransferases (MGMT) Promoter Subform Gliomas

**Fig. 1:**
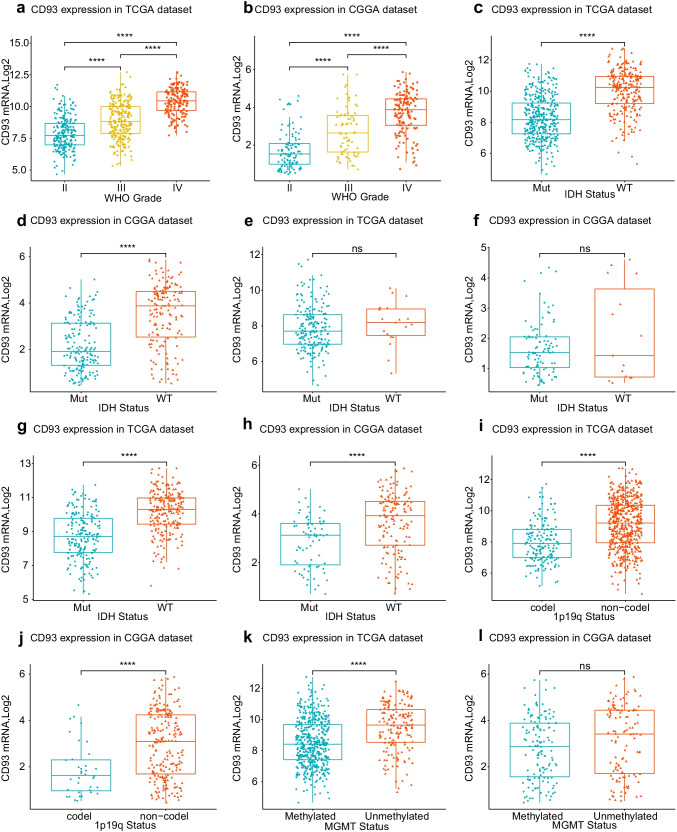
CD93 expression patterns for gliomas among various WHO grades (**a**, **b**), isocitrate dehydrogenase forms (all grades (**c**, **d**), low grades (**e**, **f**), high grades (**g**, **h**)), and 1p/19q-codeletion statuses (**i**, **j**) and O^6^-methylguanine-methylguanine-deoxyribonucleic acid methyltransferases promoter statuses (k, l). CD93 is markedly upregulated among high grades, isocitrate dehydrogenase wild form, and non-codeleted 1p19q subform along with unmethylated O.^6^-methylguanine-deoxyribonucleic acid methyltransferase promoters subform gliomas. * *P* value below 0.05, ** *P* value below 0.01, *** *P* value below 0.001, **** *P* value below 0.0001

First, we studied the corresponding RNA-seq information from these glioma cases in both sets to investigate the CD93-expression status. CD93 was remarkably overexpressed in gliomas classified as high WHO grades, particularly among patients with GBM in both cohorts (Fig. 1a, b). Moreover, the status of IDH mutation, 1p19q-codeletion, and MGMT methylation have already been verified to be essential in regard to defining glioma types and predicting the survival of patients (Louis et al. 2021). Thus, we compared the variance in the mRNA levels of CD93 among the molecular pathological types of gliomas. Our analysis results revealed notably higher levels of CD93 expression among IDH wild forms, non-codeleted 1p19q subforms, and unmethylated MGMT promoter subforms in comparison to levels in gliomas of the IDH mutation subtype (Fig. 1c, d for total WHO grades; e, f for low WHO grade; g, h for high WHO grades) and the codeleted 1p19q subform (Fig. 1i, j) in combination with methylated MGMT promoter subtype (Fig. 1k, l) according to the TCGA and CGGA databases. Therefore, the above results revealed that CD93 is overexpressed among these poor molecular pathological subtypes, and this expression status acts as an adverse biomarker for therapy reactivity and overall prognosis of glioma.

### High Expression of CD93 Relates to a Worse Prognosis for Patients with Glioma

**Fig. 2:**
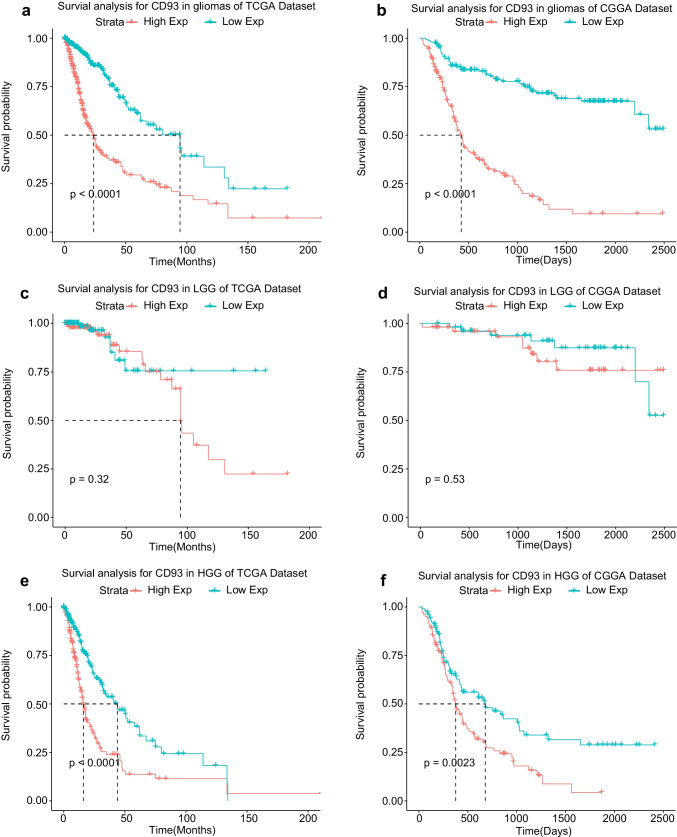
Kaplan–Meier survival curvilinear analysis for CD93 among patients with overall grades (**a**, **b**), low grade (**c**, **d**), and high grade (**e**, **f**) gliomas. Highly-expressed CD93 is related to worse prognosis for patients with glioma, particularly for patients with high grades gliomas

**Fig. 3:**
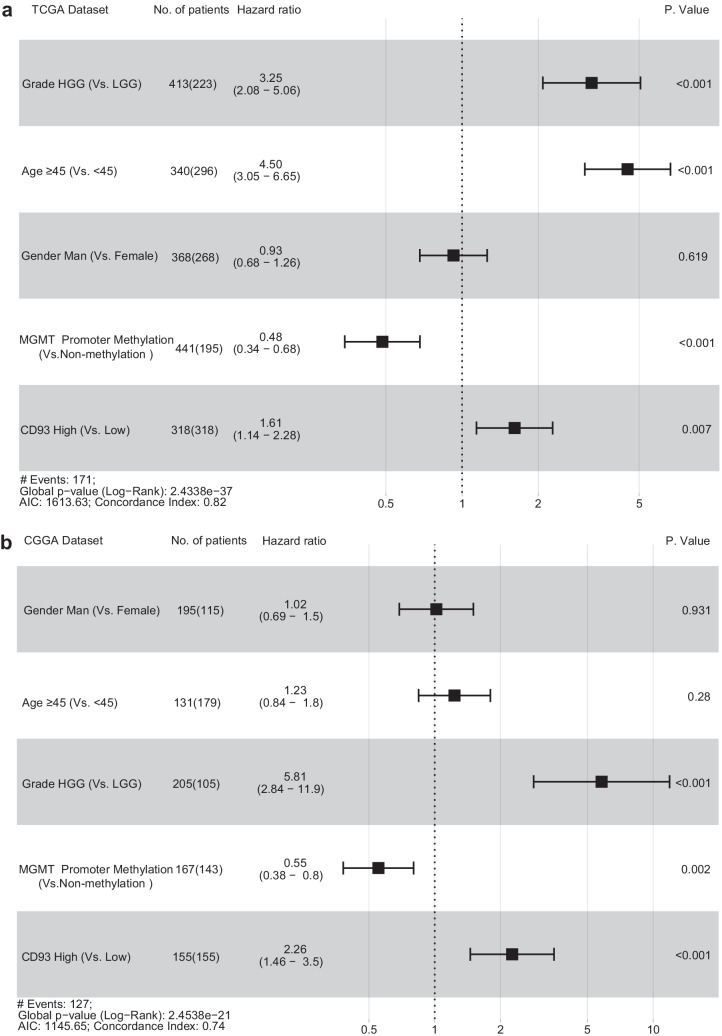
Multivariable Cox analyses of CD93 among glioma patients (**a**, **b**). High expression of CD93 is a distinct prognostic variable for patients with gliomas compared to gender, age, WHO grades and O^6^-methylguanine-deoxyribonucleic acid methyltransferases promoter statuses

These findings demonstrated that CD93 may provide a possible biomarker for malignant gliomas. Subsequently, we explored the relationship between CD93 and the prognosis of glioma patients. First, we plotted the Kaplan–Meier models using the survival statistics from glioma samples from the TCGA and CGGA sets (Fig. 2a, b), and those patients with highly expressed CD93 exhibited significantly shorter overall survival (OS) time (*P* < 0.0001). Second, we separately analyzed the impact of CD93 on the OS rate for patients with low-grade gliomas (LGG) (Fig. 2c, d) as well as patients with HGG to avoid the effects of heterogeneous differences in various tumoral grades, and we determined that the impact of CD93 on OS was more apparent for HGG patients (Fig. 2e, f). Moreover, we further investigated the role of CD93 in the overall prognosis of glioma patients interconnected with certain major clinical variables such as sex, age, WHO grades, and MGMT promoter methylation status (Fig. 3a, b). The above survival analyses using multivariate COX statistics further confirmed that high expression of CD93 is a risk factor and also an adverse marker that is correlated with worse survival in glioma patients.

### CD93 is Remarkably Upregulated in Mesenchymal Subtype Glioma and is Applicable for Estimating the Mesenchyme Subform

**Fig. 4:**
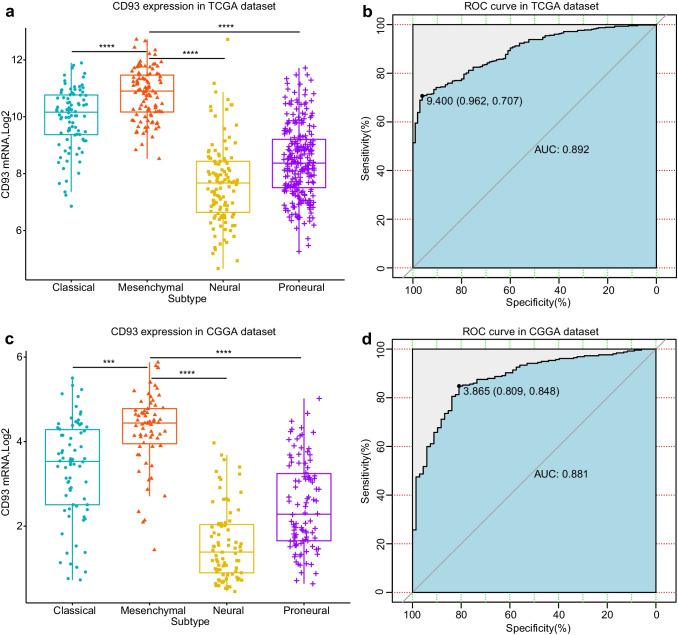
Relation between CD93 expression and TCGA subtypes of glioma. CD93 is indicated to be markedly upregulated in the mesenchyme subtype of both sets (**a**, **c**). Receiver operation characteristics analysis revealed the favorable applicability and accuracy of CD93 in estimating mesenchyme subform gliomas (**b**, **d**). * P value below 0.05, ** *P* value below 0.01, *** *P* value below 0.001, **** *P* value below 0.0001

Throughout the years, the TCGA system has classified gliomas as mesenchymal subforms along with three other molecule-based subforms (Verhaak et al. 2010). This categorization was verified as consequential to patient survival duration, particularly the mesenchyme subform that represented adverse progression together with worse survival of tumors (Ma et al. 2021). Subsequently, we studied the relationship between CD93 expression and the four subtypes in the two datasets (Fig. 4). CD93 was markedly upregulated in the mesenchymal subtype in both sets (Fig. 4a, c). Next, receiver operating characteristics analysis was utilized to estimate the favorable applicability of CD93 in estimating mesenchyme subform gliomas. For the TCGA database (Fig. 4b), the area under the curve reached 0.892 when CD93 predicted gliomas of the mesenchyme subform. Meanwhile, the corresponding specificity was 96.2%, and the sensitivity was 70.7% with an optimal cut-off value of 9.400. Similarly, the area under the curve reached 0.881 for the CGGA database (Fig. 4d), and the corresponding specificity and the sensitivity were 80.9% and 84.8% with an optimal cutoff value of 3.865. The above statistics revealed the favorable applicability and accuracy of CD93 in estimating mesenchyme subform gliomas.

### CD93 is Linked to Glioma-associated Immunizing Responses

**Fig. 5:**
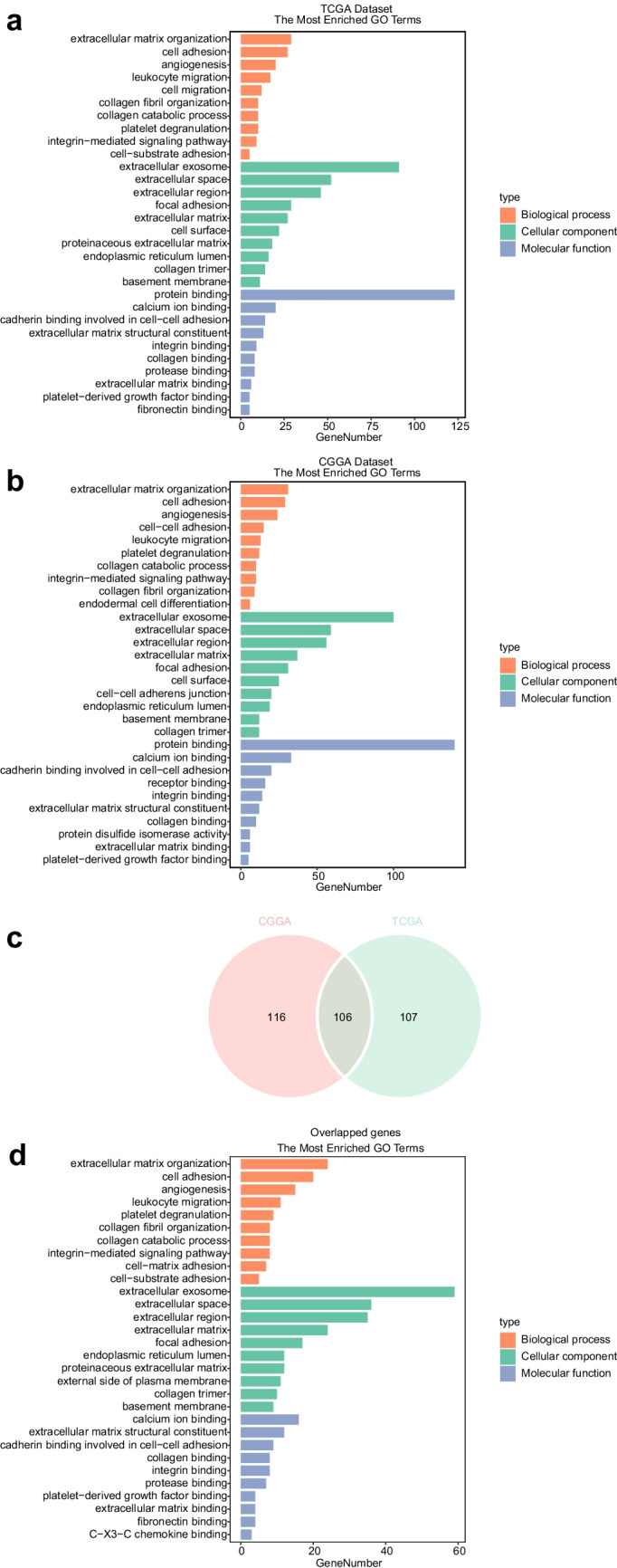
Gene ontology analysis of CD93 in gliomas among the TCGA set (**a**), the CGGA set (**b**), and the 106 overlapped genes of both sets (**c**, **d**). CD93 is closely involved in glioma-associated immune responses such as leukocyte migration, predominantly in extracellular space or on cell membranes as extracellular exosomes and membrane components

For the purpose of deeply exploring these characteristics along with the biological functions of CD93, we sequenced CD93-related genes according to Spearman correlation analysis (Supplementary Table S1). For the TCGA dataset, we finally filtered out 213 relevant genes for those with absolute correlation coefficient values of greater than 0.7 (*P* < 0.05), and among these, 196 exhibited a positive correlation with CD93, while 17 exhibited a negative correlation. Using the same criterion, 222 relevant genes were filtered out for the CGGA dataset, and among these, 211 exhibited a positive correlation with CD93, while 11 exhibited an opposite correlation. Subsequent gene ontology analyzation of these genes was conducted using the DAVID website (Fig. 5a, b). CD93-involved biological procedures primarily include angiogenesis, leukocyte migrations, integrin-mediated signaling pathway, platelet degranulation, endodermal cell differentiation, cell adhesions and migrations, cell-substrate adhesions, extracellular matrix organization, collagen catabolic process and other biological processes. Regarding cellular components, CD93 chiefly works on cell surface and basement membranes, or in extracellular exosomes, extracellular region, ECM, focal adhesion and cell–cell adherens junctions. Several major molecular functions of CD93 include protein and calcium ion binding, receptor and ECM binding, integrin and protease binding, ECM structural constituent, collagen and fibronectin binding, platelet-derived growth factor binding and protein disulfide isomerase activity. These findings were consistent for both the TCGA and CGGA cohorts. Furthermore, gene ontology analysis of these 106 correlated genes from both datasets was performed to provide an additional validation as presented in Fig. 5c and d as well as Supplementary Table S2. What is interesting among these results is that CD93 involves in glioma-associated immune responses such as leukocyte migration, which may play a role in the immune microenvironment of gliomas.

**Fig. 6:**
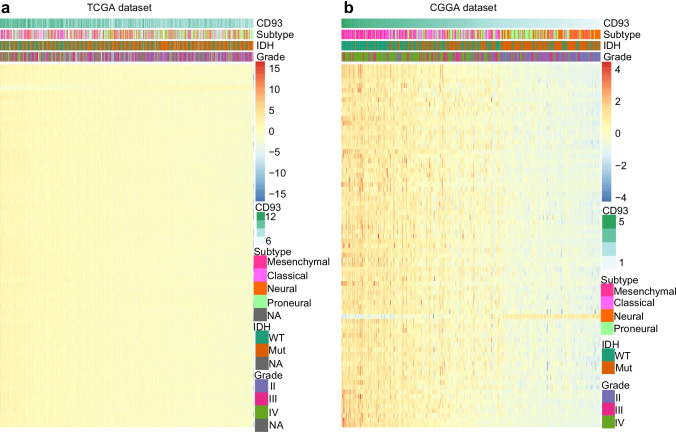
Heatmap analysis of correlations among immunogene subsets and CD93 for glioma (**a**, **b**). Immunizing genes predominantly exhibit a positive correlation with CD93 for both datasets, thus revealing the role of CD93 in the context of glioma-associated immune response

To investigate the functions of CD93 in the context of glioma-associated immune responses, these immunogene subsets were downloaded from the AmiGO2 website. Based on these subsets, 70 immunizing genes in the TCGA dataset and also 77 genes from the CGGA set that were notably related to CD93 (|R|> 0.7, *P* < 0.05) were selected to perform heatmap analysis (Figs. 6a, b; S1a, b). A list of these genes is provided in Supplementary Table S3. Finally, we concluded that the above immunizing genes exhibit a positive correlation with CD93 in both datasets, thus further revealing the role of CD93 in glioma-associated immune responses.

### CD93 is Strongly Relevant to the Inflammation Activities of Gliomas

**Fig. 7:**
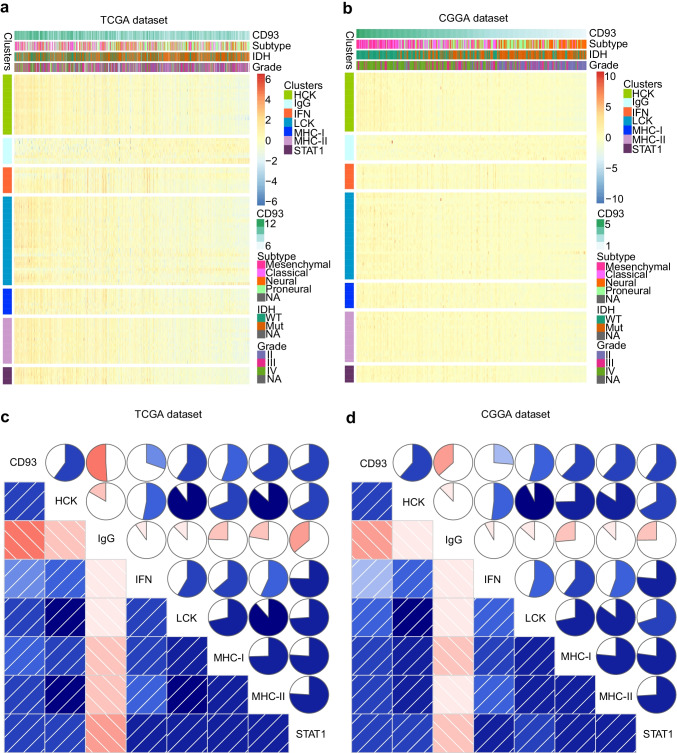
Inflammation-related functions of CD93 among gliomas. Heatmap analysis of correlations of inflammatory genes with CD93 expression (**a**, **b**). Genes set variant analysis of CD93 and inflammatory metagenes (**c**, **d**). Blue indicates positive correlation, while red indicates negative correlation. Bicolor gradation together with the circle dimension are in proportion to correlational degree. CD93 expression is significantly positively correlated with hematopoietic cell kinase (HCK), lymphocyte-specific protein tyrosine kinase (LCK), major histocompatibility complex (MHC) I, MHC II, signal transducer and activator of transcription 1 (STAT1), and interferon (IFN), while immunoglobulin G (IgG) is negatively correlated

As stated based on the above experiments, CD93 is associated with inflammatory responses in glioma. Therefore, to search for specific inflammation-related functions of CD93, we included 104 inflammation genes that could generally fall into seven metagenes (Ma et al. 2021). Supplementary Table S4 contains the specified lists of these metagenes. In the CGGA and TCGA databases, heatmap analysis (Figs. 7a, b; S3a, b) of those inflammatory metagenes demonstrated their correlations with CD93, and immunoglobulin G (IgG) metagenes exhibit an inverse connection with CD93, while the other six metagenes exhibited an opposite connection. For further confirmation, gene set variant analysis of CD93 and also the metagenes described above was conducted to plot corresponding correlograms based on Pearson correlation analysis (Fig. 7c, d). The above analyses among the TCGA and CGGA databases were highly coherent with the heatmaps. We verified that CD93 expression is significantly positively correlated with hematopoietic cell kinase (HCK), lymphocyte-specific protein tyrosine kinase (LCK), major histocompatibility complex (MHC) I, MHC II, signal transducer and activator of transcription 1 (STAT1), and also with interferon (IFN); however, the results for IgG were the opposite.

### Associations’ Analyses Examining CD93 in Relation to Glioma-infiltrating Immunocytes

**Fig. 8:**
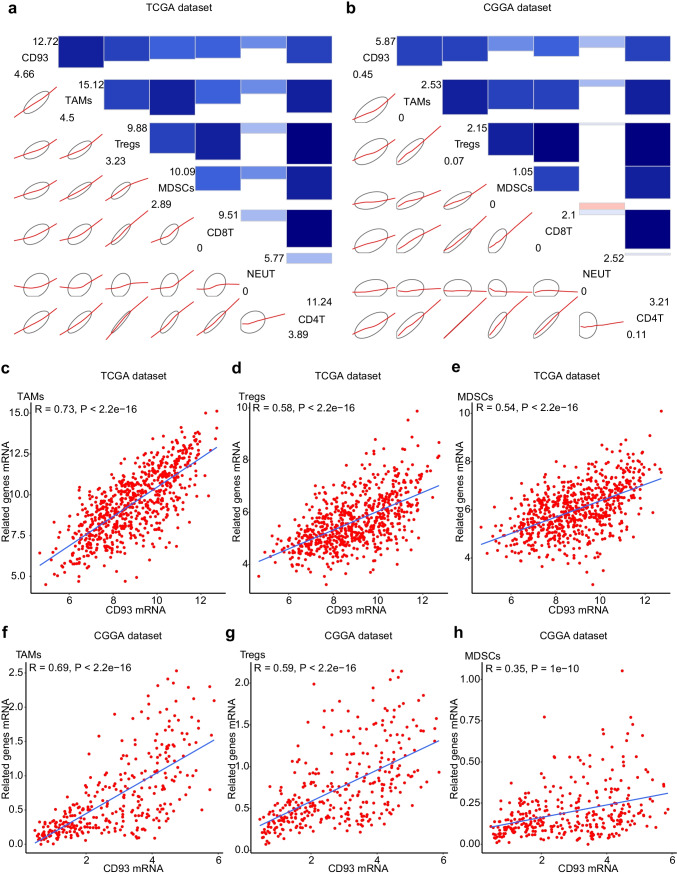
Associations analyses of CD93 and gliomas-infiltrating immunocytes. Corrgram analyses visualizing associations of CD93 with six major tumor-infiltrating immunocyte subpopulations (**a**, **b**). Blue indicates positive correlation, while red indicates negative correlation. Bicolor gradation together with the circle dimension are in proportion to correlation degree. The leading diagonal contains the minimum and maximum values of the variables. Pearson associations analyses of CD93 and tumor-associated macrophages (TAMs) (**c**, **f**), regulatory T lymphocytes (Tregs) (**d**, **g**) and myeloid-derived suppressor cells (MDSCs) (**e**, **h**) among the two datasets. Cases of glioma are displayed as dots, and regression analyzing lines are added into corresponding points in the diagram

The infiltration of immunocytes into tumors has been verified as a key component of the immunosuppressive microenvironment and also the invasive processes of malignant gliomas (Kim et al. 2021). Thus, further analyses examining the association between CD93 and glioma-infiltrating immunocytes are indispensable. We selected six major tumor-infiltrating immunocyte subpopulations for analysis (Supplementary Table S5). Later, corrgram analyses were performed to visualize associations of CD93 with the above immunocyte subpopulations among the TCGA and CGGA datasets (Fig. 8a, b), and our results indicated that the infiltration degrees for most immunocytes were positively correlated with CD93 expression. Moreover, Pearson association analyses indicated that CD93 was positively correlated with immunosuppressive subsets such as tumor-associated macrophages (TAMs) (Fig. 8c, f), regulatory T lymphocytes (Tregs) (Fig. 8d, g), and myeloid-derived suppressor cells (MDSCs) (Fig. 8e, h) among those two datasets. For TAMs, the correlation coefficient values for the TCGA and CGGA sets were 0.73 and 0.69. For Tregs, the correlation coefficient values for the TCGA and CGGA sets were 0.58 and 0.59. For MDSCs, the correlation coefficient values for the TCGA and CGGA sets were 0.54 and 0.35.

## Discussion

Although a number of clinical trials examining glioma therapies have not led to any satisfying advances based on targeted therapies, novel therapeutic strategies integrating adequate practical biomarkers inextricably linked to glioma clinicopathology are encouraging in regard to surmounting existing treatment woes (Ma et al. 2021; Tan et al. 2020). This study demonstrates that CD93 is markedly associated with adverse pathology types, unfavorable survival, and immunosuppressive immunocyte infiltration degrees among gliomas, and this suggests that CD93 may serve as a practicable marker and a promising target for glioma-based precise diagnosis and therapeutic strategies.

Earlier reports demonstrated that CD93 in the endotheliocytes of GBM was selectively and stably expressed compared to expression in normal brain tissue (Langenkamp et al. 2015; Lugano et al. 2018; Xie et al. 2021). CD93 predominantly located in the core and the invading margins of GBM (Langenkamp et al. 2015), which has been verified as one of the top ten enrichment genes among ECs in the GBM core (Xie et al. 2021). Regarding the heterogeneity of glioma, we also analyzed the anatomic structures RNA-Seq data of 122 RNA samples in Ivy Glioblastoma Atlas Project (IVY GAP (http://glioblastoma.alleninstitute.org/)) (Puchalski et al. 2018), and we found that CD93 was significantly upregulated in glioma core area compared with peripheral area (Fig. S3). We observed that CD93 expression levels are markedly upregulated in glioma patients with high grade tumors. Similarly, analysis of molecular pathological subforms displayed notably higher levels of CD93 expression among IDH wild-type, non-codeleted 1p19q, and unmethylated MGMT promoter subforms. Additionally, CD93 has been demonstrated to exhibit favorable applicability in regard to estimating mesenchyme subform gliomas. Based on the recognized clinical significance of these molecular pathological subforms for glioma, glioma patients with elevated CD93 expression in tumor tissue may possess a high probability of neoplasm invasion, local recurrence, and treatment insensitivity. Subsequent analyses of survival identified high-expression CD93 as a distinct prognostic variable for patients with gliomas, while earlier reports have displayed similar findings in the context of HGG (Langenkamp et al. 2015), nasopharyngeal carcinoma (Bao et al. 2016) and colon cancer (Olsen et al. 2015) but not in overall gliomas. CD93 have been reported to be important for the growth and invasion of GBM (Langenkamp et al. 2015; Lugano et al. 2018). CD93 was actively involved in the regulation of pathologic angiogenesis, vessel architecture and vascular function for gliomas (Langenkamp et al. 2015; Sun et al. 2021b; Xie et al. 2021). Highly expressed CD93 contributed to angiogenesis through promoting tubular morphogenesis, cytoskeletal reconstruction, cell junctions formation, adhesion and migration of endothelial cells in HGG (Langenkamp et al. 2015). CD93 deficiency could significantly induce defects in interendothelial junctions, increased permeability and decreased perfusion of the glioma vasculatures (Langenkamp et al. 2015). CD93 knockdown markedly reduced the angiogenesis and tumor growth of GBM in vivo and in vitro (Langenkamp et al. 2015). CD93 domains promote functional angiogenesis mainly through the PI3K/Akt/eNOS and ERK1/2 pathways (Kao et al. 2012). Meanwhile, CD93 promoted the tumoral neovascularization of high permeability through its interaction with insulin-like growth factor binding protein 7(IGFBP7) in GBM (Pen et al. 2011; Sun et al. 2021b). Tumor angiogenesis can induce immunosuppression in glioma with abnormal vasculatures (Jain 2005; Pen et al. 2008), restricted perfusion (Jain et al. 2007), hypoxic and acidic TME (Huang et al. 2013). Above factors remarkably impair the lymphatic flow and compromise the cytotoxic functions of infiltrating immune effector cells (Huang et al. 2013; Jain 2005; Noman et al. 2015) through down-regulating adhesion molecules including intercellular adhesion molecule 1 and vascular cell adhesion molecule 1 (Bouzin et al. 2007), up-regulating immune checkpoints (Noman et al. 2014, 2015) and activating multiple immune-suppressive growth factors or cytokines (e.g., VEGF, TGF-β) (Huang et al. 2013), which can also promote the recruitment of Tregs and MDSCs and drive TAMs to their suppressive form (Chanmee et al. 2014; Domènech et al. 2021; Huber et al. 2017). Also, the function of tumor ECs is largely immunosuppressive, maintained by tumor cells through paracrine mechanisms (Motz and Coukos 2013; Mulligan et al. 2009; Mulligan and Young 2010), which may hinder the maturity, and thus the efficacy, of antigen presentation by dendritic cells (DCs) (Huang et al. 2013; Peterson et al. 2016). Immunosuppressive cells can in turn promote tumor angiogenesis (Rivera et al. 2015; Shojaei et al. 2007). The resident microglia and TAMs promote glioma vascularization by the overexpression of multiple pro-angiogenics; of special relevance is the CXCL2 pathway (Brandenburg et al. 2016; Grégoire et al. 2020). Tregs could directly promote tumor angiogenesis via secreting VEGF and recruiting endothelial cells (Facciabene et al. 2012, 2011), or indirectly through inhibiting Th1 cell activation and polarizing TAMs into the M2-like phenotype (Mantovani et al. 2013). MDSCs directly promote tumor angiogenesis by producing VEGF, FGF2, Bv8, and matrix metalloproteinase (Bruno et al. 2019; Lee et al. 2020). Moreover, CD93 was also involved in the matrix organization for ECs in vascularization processes of gliomas (Lugano et al. 2018). CD93 activates β1 integrin signaling and organizing of fibronectin fibrillogenesis in HGG (Lugano et al. 2018). The interaction between CD93 and multimerin-2 (MMRN2) delivers integrin-dependent signals to regulate Src activation, fibronectin deposition, endosialin loss and prevent the development of fibrosis in ECM during glioma angiogenesis (Liang et al. 2020; Lugano et al. 2020, 2018). CD93-mediated neovascularization and the remodeling of blood brain barrier (BBB) provided ideal nutritional support for tumor cells, which formed an invasive niche suitable for the progression of HGG (Langenkamp et al. 2015; Xie et al. 2021). CD93 also acts as an important component in glucometabolic regulation for tumor ECs of high glycolysis in GBM (Strawbridge et al. 2016; Xie et al. 2021). The above researches further sustain our findings. These studies further support the findings of this study. Consequently, CD93 could be regarded as a practical marker for glioma-based evaluation of molecular pathological subforms and also of long-term survival.

In regard to these roles and the biologic processes mediated by CD93 in the context of glioma, besides angiogenesis and ECM organization, we found that CD93 chiefly involved in leukocyte migrations, integrin-mediated signaling pathway, platelet degranulation and others. Subsequent association analysis documented the predominant role of CD93 among glioma-relevant immunobiological processes and also inflammatory responses. Interestingly, we found that CD93 is negatively correlated with IgG. CD93-induced abnormal leaky and tortuous tumor vasculatures (Caro-Maldonado et al. 2014), ineffective perfusion of glioma tissue (Caro-Maldonado et al. 2014), tumor necrosis and hypoxia (Guyon et al. 2021; Sattiraju and Mintz 2019), immunosuppressive molecules production (Munn and Jain 2019), down-regulated adhesion molecules (Fontana et al. 1992; Munn and Jain 2019; Sun et al. 2021b), up-regulated immune checkpoints (Noman et al. 2014, 2015; Okazaki et al. 2001; Thibult et al. 2013), and increased immunosuppressive cells (Wang et al. 2018; Zhao et al. 2006) may synergistically lead to reduced B-cell proliferation, impaired B-cell function, and decreased IgG production. Immunocytes infiltrating together with immunosuppressive events have been demonstrated to be important for invasion and also for therapeutic insensitivity in glioma (Ma et al. 2021; Xu et al. 2020); however, little is known regarding the specific role of CD93 in the above activities. Moreover, association analyses examining CD93 and glioma-infiltrating immunocytes indicated that the degree of infiltration of most immunocytes exhibited positive correlations with CD93 expression, particularly in immunosuppressive subsets such as TAM, MDSCs, and Tregs. It was also reported that Tregs could suppress B-cell proliferation by inducing granzyme-dependent cell death (Zhao et al. 2006) and MDSCs could impair B-cell function of antibody production through the secretion of IL-7 (Wang et al. 2018), which similarly explains the negative correlation between CD93 and IgG. We also preliminarily explored CD93 expression among different cells in various single-cell RNA-seq glioma datasets via single-cell TIME (scTIME) portal (Hong et al. 2021), Tumor Immune Single-cell Hub (TISCH) (Sun et al. 2021a), Single Cell Portal (https://singlecell.broadinstitute.org/single_cell) and Cancer Single-cell Expression Map (CancerSCEM) (Zeng et al. 2022). After analyzing GSE131928 (Fig. S4a) (Neftel et al. 2019), GSE84465 (Fig. S4b) (Darmanis et al. 2017), GSE138794 (Fig. S4c) (Wang et al. 2019), GSE139448 (Fig. S5a) (Wang et al. 2020), GSE103224 (Fig. S5b) (Yuan et al. 2018), GSE102130 (Fig. S5c) (Filbin et al. 2018), GSE70630 (Fig. S5d) (Tirosh et al. 2016), GSE148842 (Fig. S5e) (Zhao et al. 2021), GSE131928 (Fig. S6a) (Neftel et al. 2019), GSE89567 (Fig. S6b) (Venteicher et al. 2017), GSE141946 (GBM-009–01-1C) (Fig. S6c) (Jacob et al. 2020), GSE141946 (GBM-009–04-1C) (Fig. S6d) (Jacob et al. 2020), GSE139448 (GBM-010–02-1A) (Fig. S6e) (Wang et al. 2020), GSE139448 (GBM-010–03-1A) (Fig. S6f) (Wang et al. 2020), GSE84465 (GBM-011–03-1B) (Fig. S6g) (Darmanis et al. 2017) and GSE84465 (GBM-011–02-1B) (Fig. S6h) (Darmanis et al. 2017), we found that CD93 was highly expressed in TAMs of glioma for most single-cell RNA-seq datasets. These inspiring explorations reveal the potential involvement of CD93 in glioma-connected immunosuppression along with invasion. Previous studies confirmed that CD93 formed granular membranes and involved in NEUT degradating, activating, as well as immune responses in glioma microenvironments (Li et al. 2020). CD93 was identified as a vital regulator of the CNS autoimmune activities and inflammations (Griffiths et al. 2018; Liu et al. 2014). The knock-out of CD93 induced obvious down-regulation of numerous immune and inflammatory genes in the brain (Liang et al. 2020). Recent researches have revealed the crucial part of CD93 in promoting inflammation responses (Nativel et al. 2019; Shehata et al. 2022). Moreover, it has been proved that CD93 was actively correlated with leukocytes infiltration for several inflammation models (Greenlee-Wacker et al. 2011; Harhausen et al. 2010). For experimental autoimmune encephalomyelitis mice, these infiltrating microglial cells or monocytes with overexpressed CD93 tend to cause T cells apoptosis, which greatly immunosuppressed functional CD4^+^ and CD8^+^ T cells (Griffiths et al. 2018; Zhu et al. 2007). For mice models with peritonitis, CD93 was verified to adjust the recruiting, migrating and adhesion processes of leukocytes (Greenlee-Wacker et al. 2011). In addition, released sCD93 in serum could also respond to inflammation and immunity stressors as well as angiogenesis transmitters (Strawbridge et al. 2016). Membrane-related CD93 adjusts complement activating, leukocytes extravasating from postcapillary venules, macrophages phagocytosis and elimination of apoptosis cells (Greenlee et al. 2009; Greenlee-Wacker et al. 2011; Nativel et al. 2019; Norsworthy et al. 2004). CD93 also engenders the differentiation of monocytes to macrophage‐like cells and involves in the maturation of B cells and the survival of CD4^+^ natural killer T cells (Jeon et al. 2010; Nativel et al. 2019; Zekavat et al. 2010). Thus, the involvement of CD93 in glioma-based immunological responses together with these immunosuppressive processes indicates that CD93-targeting precise therapies are within the bounds of probability, and this provides an available premise in regard to coordinating locally aberrant immunity activities, obstructing immunosuppressive invasion, and raising glioma sensitivity towards treatments (Burster et al. 2021).

Based on the expression status and survival characteristics along with the known biologic functions of CD93 among gliomas, we conceived CD93 as a practicable marker and a promising target for glioma-relevant precise diagnosis and therapeutic strategies. CD93-based diagnostic and treatment strategies have been applied to many diseases. CD93 blockade was proved to be effective in antiangiogenic therapy and vascular normalization of cancers (Iwasaki et al. 2015; Orlandini et al. 2014). For gliomas, CD93 is currently considered as a possible therapeutic target since CD93-targeted therapy could significantly inhibit the growth and vascular perfusion of glioma in vivo by decreasing tumoral angiogenesis and repairing integrity of endothelial tight junctions (Langenkamp et al. 2015). Monoclonal antibodies (mAb) targeting CD93 ectodomain have been demonstrated to restrain new vessel formation both in vitro and in vivo by inhibiting the proliferation, migration, sprouting and generation of tubular structure for tumor ECs (Orlandini et al. 2014), while have no exact side effects for vessels of normal tissues (Sun et al. 2021b). Meanwhile, anti-CD93 mAb was reported to stabilize vasculatures in animal models of pancreatic cancer and malignant melanoma to facilitate therapy responses for immunotherapeutic as well as chemotherapy (2021; Sun et al. 2021b). Vascular normalization of anti-CD93 can normalize the immunosuppressive TME and promote antitumor immunity through improving perfusion and oxygenation in tumors (Stylianopoulos and Jain 2013; Sun et al. 2021b), upregulating the expression of adhesion molecules (Fukumura et al. 2018; Shigeta et al. 2020), promoting tumoricidal immune cells infiltration and improving their function (Fukumura et al. 2018; Huang et al. 2012), reducing immunosuppressive cell levels (Zhou et al. 2020), skewing TAM polarization toward the M1 phenotype (Peterson et al. 2016), enhancing immune response by activating of DCs, cytotoxic T lymphocytes, and natural killer cells (Rolny et al. 2011). CD93 blocking was shown to increase the infiltrating levels of CD4^+^ T cells, CD8^+^ T cells, NK, NK T cells and decrease the infiltrating level of MDSCs in tumors (Sun et al. 2021b). Several studies certified the intratumoral enhanced infiltration of immune cells as a result of the adhering molecules increasing and vasculature normalization induced by CD93-blocking (2021; Hamzah et al. 2008; Huang et al. 2012; Schmittnaegel et al. 2017). Furthermore, CD93 blocking effectively elevated the proportion of effector T cells in melanoma, making tumors more sensitive to anti-PD-1/PD-L immune checkpoints treatment (Sun et al. 2021b). The blockade of CD93 pathway could also decrease intratumorally hypoxemia and improve drugs delivery through vasculatures normalizing and leakage reducing, such as the interaction blocking between CD93 and IGFBP7 with mAb remarkably enhanced the antitumoral responses to gemcitabine or fluorouracil in pancreatic carcinoma bearing mice (Sun et al. 2021b). CD93 signaling blocking with metoclopramide significantly impaired the stemness and proliferation of chronic myeloid leukemia stem cells (Riether et al. 2021). It was also verified that anti-CD93 treatments restrain glucolysis and lead to dysmetabolism in tumors (Cantelmo et al. 2016; Strawbridge et al. 2016). Noninvasive ^125^I-anti-CD93 mAb radioimmunoimaging may be used for the early diagnosis and therapy delamination of non-small cell lung cancer (Liu et al. 2019). In light of these reports and together with our observations, CD93-targeting therapy is likely to supplement the present therapeutic tactics for glioma, whether administered singly or in combination with immunotherapies, and it could also be used in the context of anti-angiogenesis therapeutics. Nevertheless, further studies with single-cell RNA sequencing are currently in progress to optimize the limitations of multicellular level analysis for TCGA and CGGA datasets, which can also be used to deeply explore the expression pattern and potential mechanism of CD93 among various cell types in the immunosuppressive microenvironment of glioma. Elucidating the specific mechanism underlying the function of CD93 in glioma-associated immunosuppression remains a challenge. In the future, CD93 is expected to be applied for molecule-integrated diagnosis, comprehensive therapeutics, and fluorescence molecule imaging during operations and in the construction of targeted drug carriers for glioma.

## Conclusions

Briefly, our explorations primarily investigated CD93 expression patterns, biological functions, and clinical value in the context of glioma. Here, we determined the correlations of CD93 high expression levels with pernicious pathology types, unsatisfactory survival, neoplasm-infiltrated immunocytes, and immunosuppressive processes among gliomas, thus identifying CD93 as an encouraging marker and likely target for glioma-based precise diagnosis, therapies, and prognosis evaluations. We anticipate that CD93-targeting treatments, either individual or in combination with comprehensive therapies, will become a consequential tactic for combined and individual precise therapies for glioma.

## Supplementary Information

Below is the link to the electronic supplementary material.

Supplementary file1 (EPS 14062 KB)

Supplementary file2 (EPS 18914 KB)

Supplementary file3 (EPS 1107 KB)

Supplementary file4 (EPS 18794 KB)

Supplementary file5 (EPS 2669 KB)

Supplementary file6 (EPS 2888 KB)

Supplementary file7 (PDF 182 KB)

Supplementary file8 (PDF 139 KB)

Supplementary file9 (PDF 153 KB)

Supplementary file10 (PDF 155 KB)

Supplementary file11 (PDF 153 KB)

## Data Availability

The datasets generated during and/or analysed during the current study are available from the corresponding author on reasonable request.

## Abbreviations

CNS: Central nervous system
TTFs: Tumor treating fields
HGG: High-grade glioma
ECs: Endothelial cells
BBB: Blood brain barrier
TME: Tumor microenvironment
GBM: Glioblastoma
TCGA: The Cancer Genome Atlas
CGGA: Chinese Glioma Genome Atlas
RNA-seq: Ribonucleic acid sequencing
IDH: Isocitrate dehydrogenases
MGMT: O^6^-methylguanine-DNA methyltransferases
LGG: Low-grade glioma
OS: Overall survival
ROC: Receiver operating characteristic
AUC: Area under the curve
GO: Gene ontology
ECM: Extracellular matrix
IFN: Interferon
Tregs: Regulatory T cells
TAMs: Tumor-associated macrophages
MDSCs: Myeloid-derived suppressor cells
NEUT: Neutrophils
DCs: Dendritic cells
