# Supplementary material for: CD93 is Associated with Glioma-related Malignant Processes and Immunosuppressive Cell Infiltration as an Inspiring Biomarker of Survivance
Source: J Mol Neurosci. 2022 Aug 25;72(10):2106–24. doi: 10.1007/s12031-022-02060-4 (PMC9596571; doi:10.1007/s12031-022-02060-4)
Supplement: Supplementary file 7 — Supplementary file7 (PDF 182 KB) [file 12031_2022_2060_MOESM7_ESM.pdf]

Title: CD93 associates with the pernicious processes, immunosuppressive immunocytes infiltrating and survivance for glioma patients as an inspiring marker.
Journal Name: *Journal of molecular neuroscience*.
Authors: Kaiming Ma[1], Suhua Chen[1], Xin Chen[1,2], Xiaofang Zhao[1], Jun Yang[1,2*]
Correspondence affiliation: [1] Department of Neurosurgery, Peking University Third Hospital, Beijing, China. [2] Center for Precision Neurosurgery and Oncology of Peking University Health Science Center, Beijing, China.
Correspondence e-mail address: yangjbysy@bjmu.edu.cn.

Supplementary Table S1. Genes associated with CD93 in TCGA and CGGA datasets.
There are 213 related genes from TCGA database (196 positively corelated genes, 17 negatively corelated genes) and 222 related genes from CGGA database (211 positively corelated genes, 11 negatively corelated genes).

TCGA Dataset		CGGA Dataset	
Genes	R	Genes	R
TOM1L2	−0.72	ENHO	−0.77
ENHO	−0.72	RASL10A	−0.74
ALDOC	−0.72	ADHFE1	−0.74
PDZD4	−0.72	GLUD1	−0.73
FAIM2	−0.72	GNAO1	−0.72
AGXT2L1	−0.72	ADARB2	−0.71
BZRAP1	−0.71	ALDOC	−0.71
PNMAL2	−0.71	SERP2	−0.71
ALDH2	−0.71	MCF2L2	−0.71
CRTC1	−0.71	LINC00263	−0.71
C10orf75	−0.71	HLF	−0.71
KIAA0427	−0.71	RPN1	0.71
PRRT1	−0.71	CAV1	0.71
SERP2	−0.71	PTGFRN	0.71
C5orf53	−0.71	CREB3L2	0.71
NDRG2	−0.71	ADAM9	0.71
HDAC11	−0.71	ITPKC	0.71
ZDHHC5	0.71	PTX3	0.71
PPIL5	0.71	CFI	0.71
PLOD1	0.71	TMEM173	0.71
MGAT2	0.71	VIM	0.71
SPPL2A	0.71	LY96	0.71
DSE	0.71	ACTN4	0.71
IL4R	0.71	RARS	0.71
SVIL	0.71	S100A11	0.71
TRAM1	0.71	PRDX4	0.71
SLC39A1	0.71	REEP4	0.71
ELF4	0.71	ADAM19	0.71
PTGER4	0.71	GJA4	0.71
KDELR2	0.71	PELO	0.71
ACTN1	0.71	CMTM6	0.71
CISH	0.71	ACTA2	0.71
PTPN7	0.71	HLA−A	0.71
RBMS1	0.71	TRPC6	0.71
VEGFA	0.71	CFH	0.71
TYMP	0.71	ANXA1	0.71
STT3A	0.71	ADM	0.71
C3orf64	0.71	TNKS1BP1	0.71
HLA−DRA	0.71	EDEM1	0.72
GJA4	0.71	RCN3	0.72
GLT25D1	0.71	PDIA5	0.72
ADAM9	0.71	PDGFRB	0.72
CSDAP1	0.71	ARPC5	0.72
PODXL	0.71	GPR124	0.72
COL5A2	0.71	LAMB2	0.72
TNFAIP3	0.71	TMEM37	0.72
CAV1	0.71	ENTPD7	0.72
MSN	0.71	HEXB	0.72
TMEM71	0.71	PVRL2	0.72
OSTC	0.71	NOTCH3	0.72

TMEM49	0.71	IGFBP7	0.72
TGFB1I1	0.71	TOR4A	0.72
NOX4	0.71	ARHGDIB	0.72
FOSL1	0.71	ITGA11	0.72
FLNA	0.71	MANF	0.72
PGM2	0.71	ZNF217	0.72
PLBD1	0.71	CKAP4	0.72
NCRNA00152	0.71	ELTD1	0.72
MXRA5	0.71	TM4SF1	0.72
ANXA1	0.71	C1R	0.72
RPN2	0.71	RDH10	0.72
ADPRH	0.71	MMRN2	0.73
ETV6	0.71	MYL12A	0.73
LOX	0.71	FHOD1	0.73
CTSC	0.71	LYZ	0.73
RUNX1	0.71	THBS1	0.73
ANXA2	0.71	HMOX1	0.73
CLCF1	0.71	CAPZA1	0.73
ADAM12	0.71	MSN	0.73
GPR65	0.71	EPHA2	0.73
BACE2	0.71	LINC00346	0.73
SECTM1	0.71	SHCBP1	0.73
SLC30A7	0.71	VWF	0.73
MAP2K3	0.71	IFNGR2	0.73
ATP8B1	0.71	FOSL2	0.73
ZYX	0.71	PCDH18	0.73
ARHGAP29	0.71	IQGAP1	0.73
C13orf29	0.71	RBMS1	0.73
F11R	0.71	CSDA	0.73
CFH	0.72	ARF6	0.73
ACTA2	0.72	TMOD3	0.73
MGP	0.72	JAG1	0.73
FCGR3A	0.72	ST8SIA4	0.74
EPHA2	0.72	IGFBP2	0.74
TEAD4	0.72	FRMD8	0.74
COL15A1	0.72	MGP	0.74
IGFBP7	0.72	SPRY1	0.74
LYZ	0.72	TXNDC5	0.74
ST8SIA4	0.72	HSPA6	0.74
TNFRSF10D	0.72	FAM114A1	0.74
PROCR	0.72	DDOST	0.74
CSDA	0.72	DLL4	0.74
SEMA3F	0.72	TNFRSF1A	0.74
JAG1	0.72	GRAP	0.74
ANXA2P2	0.72	OLFML1	0.74
CDH5	0.72	TNFRSF10D	0.74
AIM1	0.72	SERPINE1	0.74
GPR4	0.72	DCBLD2	0.74
SEC24D	0.72	RIPK1	0.74
MYL12A	0.72	ATF5	0.74
GLB1	0.72	PROS1	0.74
TNFRSF10C	0.72	HK3	0.74
SPRY1	0.72	FAM20C	0.75

THBS1	0.72	COL15A1	0.75
RUNX3	0.72	WDR1	0.75
FAM70B	0.72	TMEM8A	0.75
TNFRSF12A	0.72	IL10RB	0.75
AFAP1L1	0.72	ETV6	0.75
SERPINE1	0.72	TUBA1C	0.75
MS4A6A	0.72	NDUFA4L2	0.75
SEC61A1	0.72	BAK1	0.75
CHSY1	0.72	AFAP1L1	0.75
OLFML1	0.72	ACTN1	0.75
PCDH12	0.72	SNAI2	0.75
ANPEP	0.72	CHSY1	0.75
SOCS3	0.72	SOCS3	0.75
IFNGR2	0.72	ST14	0.75
CDK2	0.72	STAB1	0.75
DOK2	0.72	PLXDC1	0.75
LOC541471	0.72	VKORC1	0.75
GJC1	0.72	VMP1	0.75
FCGBP	0.72	GLA	0.76
CTHRC1	0.72	GUSB	0.76
SH2B3	0.72	ANXA2	0.76
MSR1	0.73	IFI30	0.76
CD276	0.73	HSPA5	0.76
LAMC3	0.73	CDH11	0.76
HYAL2	0.73	CHPF2	0.76
MYOF	0.73	TNFRSF12A	0.76
S100A4	0.73	PDIA3	0.76
CMTM6	0.73	MSR1	0.76
TAGLN2	0.73	BCL3	0.76
OLFML2A	0.73	TUBB6	0.76
PLAUR	0.73	CDH5	0.77
SERPINB8	0.73	CALR	0.77
KIAA0040	0.73	ARHGAP18	0.77
IFI30	0.73	PFN1	0.77
PRSS23	0.73	FAM129A	0.77
CALD1	0.73	OLFML2B	0.77
BGN	0.73	TRAM2	0.77
ACE	0.73	ZDHHC5	0.77
PCOLCE	0.73	TPM4	0.77
RAB27A	0.73	PMM2	0.78
TMEM173	0.73	PDIA4	0.78
SUSD2	0.73	HLX	0.78
ACTR3	0.74	CD276	0.78
TM4SF18	0.74	C1QTNF6	0.78
GNS	0.74	NAMPT	0.78
CD163	0.74	ITGB1	0.78
FILIP1L	0.74	TMEM255B	0.78
VWF	0.74	GRN	0.79
MCAM	0.74	VASP	0.79
VASP	0.74	ATP8B1	0.79
LOXL2	0.74	FLNA	0.79
COL5A1	0.74	MMP14	0.79
KDELR3	0.74	SEC61A1	0.79

MYO1B	0.74	SH2B3	0.79
ITGA4	0.74	PLVAP	0.79
GPX8	0.74	KDELR2	0.79
MYO1C	0.74	ANPEP	0.79
NAMPT	0.74	GNS	0.79
IGFBP4	0.74	PRSS23	0.8
TIMP1	0.74	SEC24D	0.8
NID2	0.74	LAMC3	0.8
CLIC1	0.74	EMILIN1	0.8
LRRC32	0.74	EXOC3L1	0.8
SHC1	0.74	CLIC1	0.8
EMR2	0.74	SYDE1	0.8
IKBIP	0.75	ECSCR	0.8
MYO1G	0.75	PDLIM1	0.8
CALU	0.75	COL6A2	0.8
FSTL1	0.75	PLXND1	0.8
ANGPT2	0.75	MXRA5	0.8
CASP4	0.76	SUSD2	0.8
FNDC3B	0.76	MYO1G	0.8
LAMB1	0.76	GLT25D1	0.8
FBLIM1	0.76	GPR4	0.8
LXN	0.76	TIMP1	0.81
TM4SF1	0.76	PCDH12	0.81
COL6A3	0.76	MMP11	0.81
TXNDC5	0.76	ACE	0.81
NRP1	0.76	SLC26A2	0.81
TNFAIP8	0.77	PLOD1	0.81
COL6A2	0.77	TGFBI	0.81
PECAM1	0.77	PCOLCE	0.81
PLAU	0.77	COL5A2	0.81
ITGB3	0.77	KCNE3	0.81
FAM26E	0.78	SEMA3F	0.82
FAM129A	0.78	IKBIP	0.82
SLC26A2	0.79	LXN	0.82
EHD4	0.8	MCAM	0.82
SERPINH1	0.8	HTRA3	0.82
ECSCR	0.8	ITGB3	0.82
CD248	0.8	MYH9	0.82
PDLIM1	0.8	P4HB	0.82
THBD	0.8	IGFBP4	0.83
LUM	0.81	LUM	0.83
ELTD1	0.81	NID2	0.84
COL1A1	0.81	BGN	0.84
TPM4	0.82	SRPX2	0.84
ENG	0.82	FSTL1	0.84
ITGB1	0.83	THBD	0.84
COL1A2	0.83	EHD4	0.84
LAMC1	0.83	GPX8	0.84
FN1	0.84	SERPINH1	0.84
ENPEP	0.84	ITGA4	0.85
ITGA1	0.84	LOXL2	0.86
ITGA5	0.86	LAMB1	0.87
COL3A1	0.86	PECAM1	0.88

TES	0.86
COL4A2	0.87
COL4A1	0.88
HSPG2	0.89

ENPEP	0.88
ADAM12	0.88
COL1A2	0.88
LAMC1	0.89
CD248	0.89
COL1A1	0.9
ITGA5	0.9
ITGA1	0.9
FN1	0.9
HSPG2	0.92
COL3A1	0.93
COL4A2	0.93
COL4A1	0.93
