# Supplementary material for: CD93 is Associated with Glioma-related Malignant Processes and Immunosuppressive Cell Infiltration as an Inspiring Biomarker of Survivance
Source: J Mol Neurosci. 2022 Aug 25;72(10):2106–24. doi: 10.1007/s12031-022-02060-4 (PMC9596571; doi:10.1007/s12031-022-02060-4)
Supplement: Supplementary file 8 — Supplementary file8 (PDF 139 KB) [file 12031_2022_2060_MOESM8_ESM.pdf]

Title: CD93 associates with the pernicious processes, immunosuppressive immunocytes infiltrating and survivance for glioma patients as an inspiring marker.
Journal Name: Journal of molecular neuroscience.
Authors: Kaiming Ma[1], Suhua Chen[1], Xin Chen[1,2], Xiaofang Zhao[1], Jun Yang[1,2*]
Correspondence affiliation: [1] Department of Neurosurgery, Peking University Third Hospital, Beijing, China.
[2] Center for Precision Neurosurgery and Oncology of Peking University Health Science Center, Beijing, China.
Correspondence e-mail address: yangjbysy@bjmu.edu.cn.

Supplementary Table S2. Overlapped 106 CD93-related genes of TCGA and CGGA datasets.

Overlapped related genes
ENHO
ALDOC
SERP2
ZDHHC5
PLOD1
KDELR2
ACTN1
RBMS1
GJA4
GLT25D1
ADAM9
COL5A2
CAV1
MSN
FLNA
MXRA5
ANXA1
ETV6
ANXA2
ADAM12
ATP8B1
CFH
ACTA2
MGP
EPHA2
COL15A1
IGFBP7
LYZ
ST8SIA4
TNFRSF10D
CSDA
SEMA3F
JAG1
CDH5
GPR4
SEC24D
MYL12A
SPRY1
THBS1
TNFRSF12A
AFAP1L1
SERPINE1
SEC61A1
CHSY1
OLFML1
PCDH12
ANPEP
SOCS3
IFNGR2

SH2B3
MSR1
CD276
LAMC3
CMTM6
IFI30
PRSS23
BGN
ACE
PCOLCE
TMEM173
SUSD2
GNS
VWF
MCAM
VASP
LOXL2
ITGA4
GPX8
NAMPT
IGFBP4
TIMP1
NID2
CLIC1
IKBIP
MYO1G
FSTL1
LAMB1
LXN
TM4SF1
TXNDC5
COL6A2
PECAM1
ITGB3
FAM129A
SLC26A2
EHD4
SERPINH1
ECSCR
CD248
PDLIM1
THBD
LUM
ELTD1
COL1A1
TPM4
ITGB1
COL1A2
LAMC1
FN1
ENPEP
ITGA1
ITGA5

COL3A1
COL4A2
COL4A1
HSPG2
