# Supplementary material for: CD93 is Associated with Glioma-related Malignant Processes and Immunosuppressive Cell Infiltration as an Inspiring Biomarker of Survivance
Source: J Mol Neurosci. 2022 Aug 25;72(10):2106–24. doi: 10.1007/s12031-022-02060-4 (PMC9596571; doi:10.1007/s12031-022-02060-4)
Supplement: Supplementary file 9 — Supplementary file9 (PDF 153 KB) [file 12031_2022_2060_MOESM9_ESM.pdf]

Title: CD93 associates with the pernicious processes, immunosuppressive immunocytes infiltrating and survivance for glioma patients as an inspiring marker.
Journal Name: Journal of molecular neuroscience.
Authors: Kaiming Ma[1], Suhua Chen[1], Xin Chen[1,2], Xiaofang Zhao[1], Jun Yang[1,2*]
Correspondence affiliation: [1] Department of Neurosurgery, Peking University Third Hospital, Beijing, China.
[2] Center for Precision Neurosurgery and Oncology of Peking University Health Science Center, Beijing, China.
Correspondence e-mail address: yangjbysy@bjmu.edu.cn.

Supplementary Table S3. Immune-related genes significantly related to CD93 in TCGA and CGGA datasets.

There are 70 immune-related genes in the TCGA database and 77 immune-related genes in the CGGA database which was significantly corrected with CD93.

TCGA database Genes	CGGA database Genes
ALDOC	ALDOC
SPPL2A	CAV1
IL4R	ADAM9
ELF4	PTX3
PTGER4	CFI
ACTN1	VIM
VEGFA	LY96
HLA-DRA	S100A11
ADAM9	PRDX4
TNFAIP3	CMTM6
CAV1	HLA-A
MSN	CFH
FLNA	ANXA1
PGM2	ARPC5
ANXA1	ENTPD7
ETV6	HEXB
LOX	CKAP4
CTSC	C1R
RUNX1	LYZ
ANXA2	THBS1
CLCF1	HMOX1
GPR65	CAPZA1
SECTM1	MSN
ZYX	EPHA2
F11R	IFNGR2
CFH	IQGAP1
FCGR3A	TMOD3
EPHA2	JAG1
LYZ	IGFBP2
TNFRSF10D	TXNDC5
JAG1	HSPA6
SEC24D	DDOST
GLB1	DLL4
THBS1	TNFRSF10D
RUNX3	SERPINE1
SERPINE1	RIPK1
ANPEP	PROS1
SOCS3	HK3
IFNGR2	WDR1
DOK2	IL10RB
SH2B3	ETV6
CD276	BAK1
HYAL2	ACTN1
CMTM6	SOCS3
PLAUR	GLA
IFI30	GUSB
ACE	ANXA2
RAB27A	IFI30

ACTR3	PDIA3
GNS	BCL3
ITGA4	CALR
MYO1C	HLX
LRRC32	CD276
SHC1	ITGB1
MYO1G	GRN
ANGPT2	FLNA
CASP4	MMP14
TXNDC5	SH2B3
PECAM1	PLVAP
PLAU	ANPEP
ITGB3	GNS
CD248	SEC24D
THBD	EMILIN1
COL1A1	MYO1G
ITGB1	ACE
COL1A2	ITGB3
FN1	MYH9
ITGA1	THBD
ITGA5	ITGA4
COL3A1	PECAM1
	COL1A2
	CD248
	COL1A1
	ITGA5
	ITGA1
	FN1
	COL3A1
